# Supplementary material for: Meltwater sediment transport as the dominating process in mid-latitude trough mouth fan formation
Source: Nat Commun. 2020 Sep 15;11:4645. doi: 10.1038/s41467-020-18337-4 (PMC7493956; doi:10.1038/s41467-020-18337-4)
Supplement: Supplementary file 1 — Supplementary Information [file 41467_2020_18337_MOESM1_ESM.pdf]

Supplementary Information for

Meltwater Sediment Transport as the Dominating Process in Mid-latitude Trough Mouth Fan Formation

Benjamin Bellwald*, Sverre Planke, Lukas W. M. Becker, Reidun Myklebust

*Corresponding author. Email: benjamin@vbpr.no

This PDF file includes:

Supplementary Figures 1-4

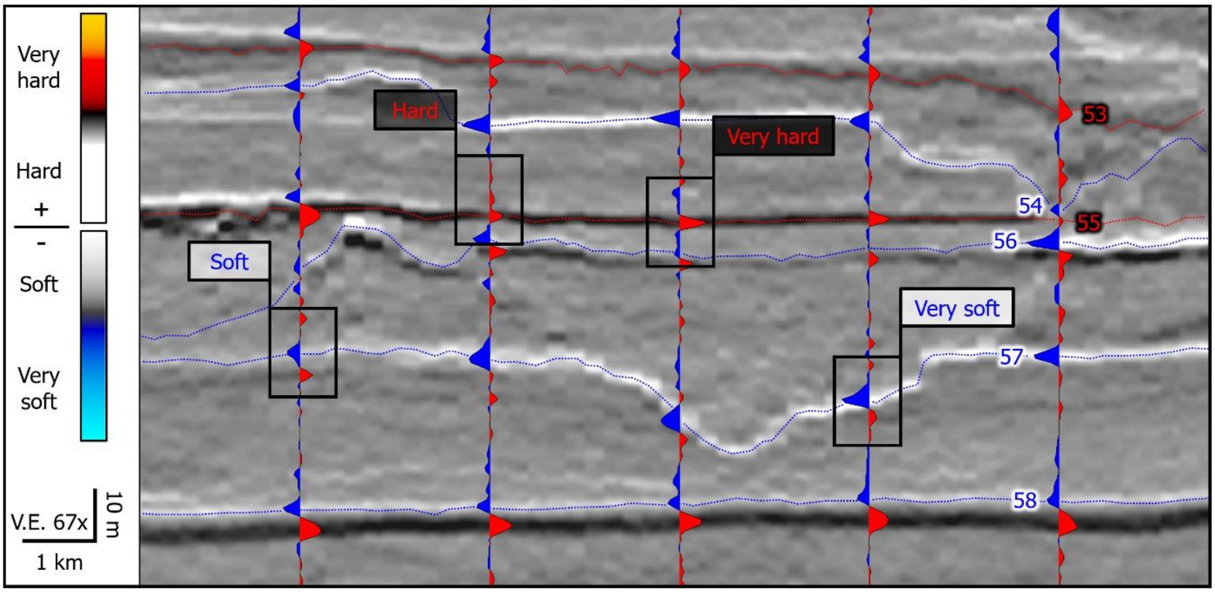

Supplementary Figure 1. Amplitude response in the shallow subsurface of the North Sea Fan. Five seismic traces with positive response (red peaks) and negative response (blue troughs) indicated. Horizons with a negative-amplitude reflection are shown as blue numbers (54, 56, 57 and 58), and horizons with a positive-amplitude reflection are shown as red numbers (53 and 55). Strength of amplitude varies along seismic reflection. The figure shows much stronger amplitudes along the reflections defining the channel-levee systems compared to the homogenous facies in between. The color bars to the left are used for imaging of geological processes in Figs. 4, 5 and 6. Seismic data courtesy of TGS.

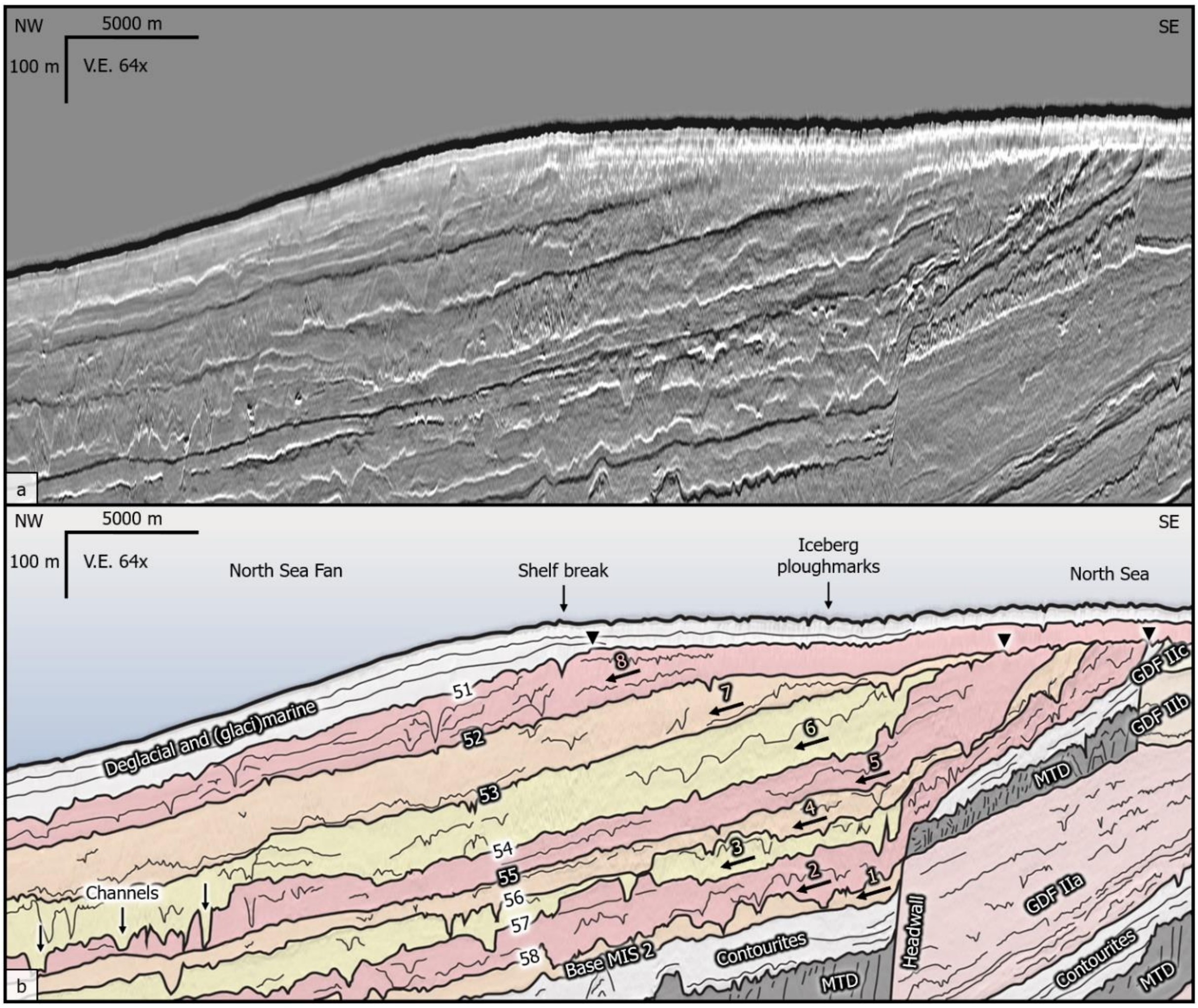

Supplementary Figure 2. High-resolution industry-standard processed 3D seismic reflection data of the North Sea Fan. **a)** Seismic profile. **b)** Interpreted seismic profile. Seismic data courtesy of TGS.

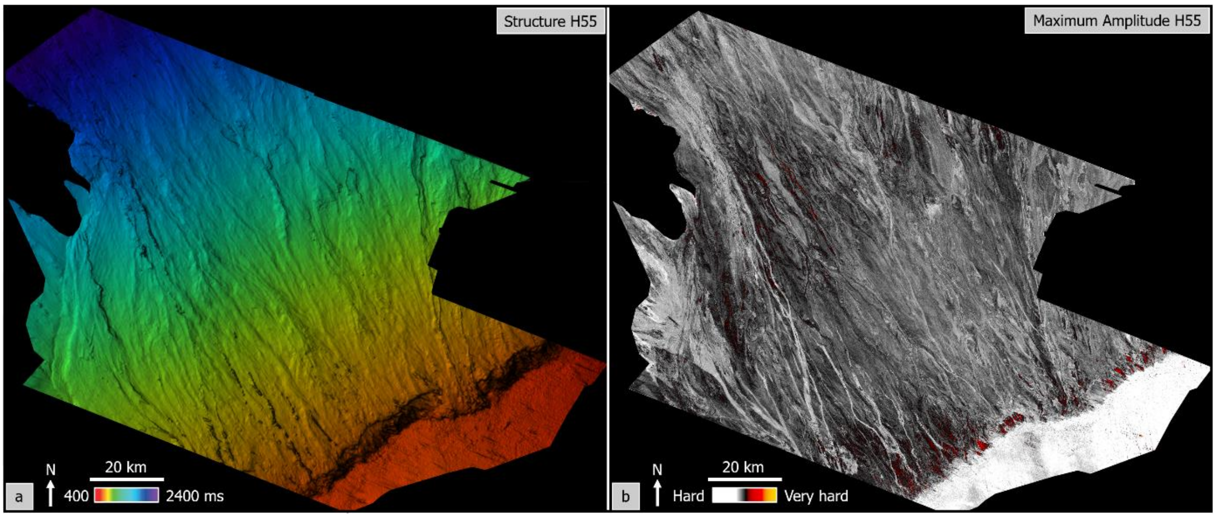

Supplementary Figure 3. Turbidite channels in the shallow subsurface of the North Sea Fan. **a)** Structure map of Horizon 55. **b)** Maximum amplitude map of Horizon 55. The grids generated of the interpreted horizon clearly show the location of the paleo-shelf break, from where southeast-northwest-oriented channels can be followed to the deeper slopes. Compared to the structure maps, the horizon amplitude attributes display the sedimentary processes related to the last shelf-edge glaciation in higher detail. Individual channel-levee systems not recognizable in structure maps are visible using seismic amplitude attributes. Maps cover the extent of AMS17 survey (shown in Fig. 2a).

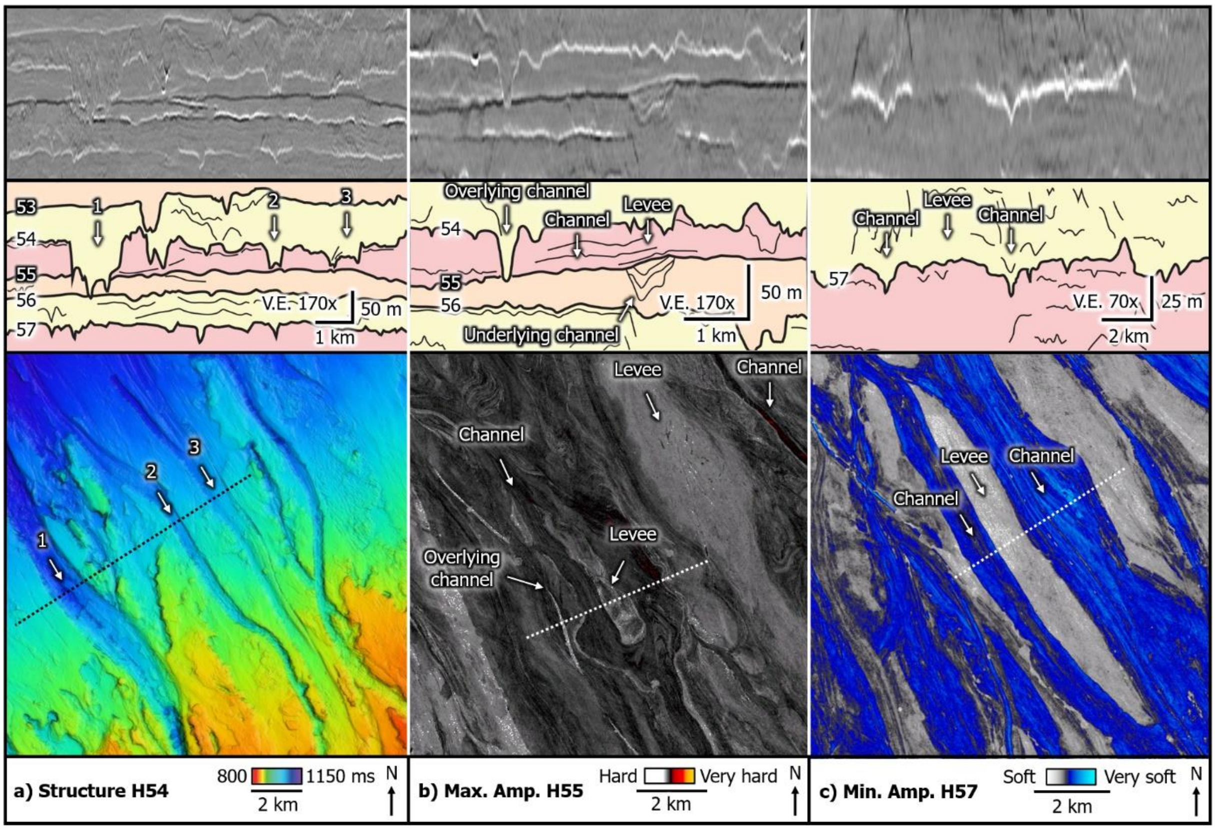

Supplementary Figure 4. Interpreted sketches of Fig. 4. Color coding of the different glacial sub-units according to Fig. 3. Seismic data courtesy of TGS.
